# Effect of *Lactobacillus casei* fermented milk on fracture healing in osteoporotic mice

**DOI:** 10.3389/fendo.2022.1041647

**Published:** 2022-10-28

**Authors:** Xing Guo, Kai Zhong, LongFei Zou, Hao Xue, ShuLing Zheng, Jiang Guo, Hui Lv, Ke Duan, DengHua Huang, MeiYun Tan

**Affiliations:** ^1^ Department of Burn and Plastic Surgery, Affiliated Hospital of Southwest Medical University, Luzhou, China; ^2^ Department of Orthopaedics, Affiliated Hospital of Southwest Medical University, Luzhou, China

**Keywords:** antibiotics, gut microbiota, probiotics, osteoporosis, fracture healing, renin-angiotensin system, RANKL pathway

## Abstract

The interaction between the gut microbiota and the host has been described experimentally by germ-free animals or by antibiotic-disturbed gut microbiota. Studies on germ-free mice have shown that gut microbiota is critical for bone growth and development in mice, emphasizing that microbiota dysbiosis may interfere with normal bone development processes. This study aimed to clarify the effect of antibiotic treatment on disturbed gut microbiota on bone development in mice and to investigate the effect of probiotic treatment on fracture healing in mice with dysbiosis. Our results showed that 4 weeks old female Kunming mice showed significantly lower abundance and diversity of the gut microbiota and significantly lower bone mineral density after 12 weeks of antibiotic treatment and significantly increased levels of RANKL and Ang II in serum (p<0.05). Mice with dysbiosis received 5 mL of *Lactobacillus casei* fermented milk by daily gavage after internal fixation of femoral fractures, and postoperative fracture healing was evaluated by X-ray, micro-CT scan, and HE staining, which showed faster growth of the broken ends of the femur and the presence of more callus. Serological tests showed decreased levels of RANKL and Ang II (p<0.05). Similarly, immunohistochemical results also showed increased expression of α smooth muscle actin in callus tissue. These results suggest that oral antibiotics can lead to dysbiosis of the gut microbiota in mice, which in turn leads to the development of osteoporosis. In contrast, probiotic treatment promoted fracture healing in osteoporotic mice after dysbiosis, and the probiotic effect on fracture healing may be produced by inhibiting the RAS/RANKL/RANK pathway.

## Introduction

Bacteria, viruses, fungi, and protozoa colonized in the gut together form the gut microbiota. In recent years, these microorganisms have been shown to have a strong association with the host, including intestinal physiology, metabolic function, immune system function, and inflammatory processes (1–4). And surprisingly, the gut microbiota has also been found to influence bone growth and development, for example, germ-free mice have lower levels of osteoclastogenic and higher bone mass (5), while male mice with intestinal infections caused by pathogenic bacteria were shown to have increased bone loss (6), and probiotic treatment reduced bone loss in ovariectomized female mice (7, 8) and type 1 diabetic male (9). These studies suggest that dysbiosis of the gut microbiota can lead to bone loss and that probiotic treatment can prevent bone loss due to some factors. However, the effect of antibiotic treatment on bone growth and development in mice remains unclear, and this effect seems to be related to the strain, sex, and age of the mice. Therefore, in this study, female Kunming mice at 4 weeks were selected for study, and antibiotic treatment was applied for 12 weeks to clarify the effect of antibiotic treatment on bone metabolism.

Oral probiotics are currently the most commonly used treatment for gut microbiota dysbiosis, and available studies have shown that probiotic therapy can alleviate multiple causes of osteoporosis, such as glucocorticoid-induced osteoporosis (10), postmenopausal osteoporosis (7, 11) and alveolar bone loss due to periodontitis (12). It is well known that osteoporosis can lead to the slow growth of fracture ends and is an important cause of delayed fracture healing. The effect of probiotic therapy on fracture healing in osteoporotic states is still unclear and deserves further study.

*Lactobacillus casei*, one of the most widely researched and used probiotics, the *Lactobacillus casei* treatment has been shown to alleviate bone loss due to a variety of factors such as type 1 diabetes (13), rheumatoid arthritis (14), ovariectomized (15), etc. Experiments have demonstrated that *Lactobacillus* fermented milk can affect bone metabolisms, such as promoting osteoblast bone formation *in vitro* and alleviating osteoporosis in spontaneously hypertensive rats and de-ovulated rats (16–18). Studies have shown that *Lactobacillus* fermented milk produces valinyl-prolinyl-proline (VPP) and the bioactive peptide isoleucine-prolinyl-proline (IPP), and in addition, these small peptides have angiotensin-converting enzyme (ACE) inhibitory activity that blocks the conversion of angiotensin I (AngI) to angiotensin II (AngII) and inhibits the breakdown of bradykinin by inhibiting ACE (16–18). Recent studies have shown that the expression of components of the renin-angiotensin system (RAS), such as renin, ACE, and Ang II receptors, are present in the local bone microenvironment and callus and are important for bone growth and development (19–21). Activation of RAS in the bone microenvironment has been shown to contribute to osteoporosis by stimulating the release of receptor activator for nuclear factor-κ B Ligand (RANKL) from osteoblasts (22–24), while inhibition of local RAS activation can alleviate bone loss and accelerate bone healing and remodeling (21, 25). Therefore, we hypothesized that dysbiosis of the gut microbiota due to antibiotic treatment could lead to osteoporosis in mice and that oral treatment with *Lactobacillus casei* fermented milk could alleviate bone loss and accelerate fracture healing in mice, and this effect might be produced by modulating the local RAS.

Herein, our results show that 12 weeks of antibiotic treatment leads to osteoporosis in mice. Oral treatment with *Lactobacillus* fermented milk significantly reduced Ang II and RANKL levels in serum, alleviated osteoporosis, and promoted fracture healing in mice.

## Materials and methods

### Animals and study design

**Figure 1:**
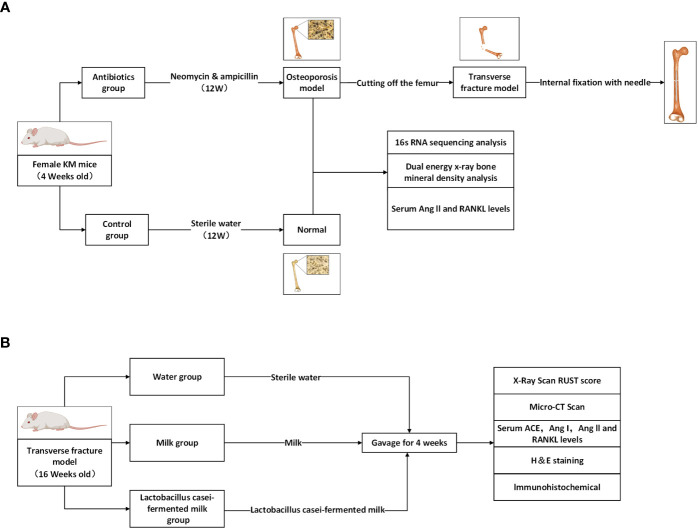
Schematic diagram of the process designed for this study. **(A)** Antibiotic feeding induced osteoporosis model establishment in mice. **(B)** Antibiotic group mice were randomly divided into 3 groups and given different interventions, and the corresponding indexes were tested after 4 weeks.

Eighty-4-week-old female Kunming mice from Chengdu Dasuo Experimental Animal Co., Ltd. were randomly divided into control (n=40) and experimental groups (n=40). Mice were kept in a special pathogen-free environment with free access to sterilized food and autoclaved water. In the experimental group, broad-spectrum antibiotics (0.5 g/l neomycin, 1.0 g/l ampicillin) were added to the drinking water starting at 4 weeks of age, and the water with or without antibiotics was renewed every two days. After 12 weeks of treatment (age: 16 weeks), the bone mineral density of the lumbar vertebrae (L3-4) of mice was measured by dual-energy X-ray scanning to clarify the effect of antibiotic treatment on bone metabolism in mice and to establish a model of osteoporosis after gut microbiota dysbiosis (**Figure 1A**). After confirming the model establishment, the mice were anesthetized by intraperitoneal injection of 1% sodium pentobarbital solution at a dose of 50 mg/Kg, and the right femur of the mice was surgically exposed to the middle segment, and the femoral stem was cut off transversely and flatly with a scalpel to cause transverse fracture of the femur. Then the femoral stem was repositioned so that both ends were fixed and the knee joint was in a flexion-neutral position, and a 1 mL sterile syringe needle was inserted into the medullary cavity parallel to the femoral stem using the center of the intercondylar fossa as the entry point to confirm that the fracture end was well aligned and the needle was firmly fixed, and the remaining needle was cut to make it completely fixed in the femur. After surgery, the experimental mice were divided into the Water group (n=11), Milk group (n=11), and *Lactobacillus casei* fermented milk group (n=11), and were gavaged with sterile water, milk and *Lactobacillus casei* fermented milk (5mL/d) for four weeks (**Figure 1B**). All animal experiments were approved by the Animal Research and Care Committee of Southwestern Medical University.

### Preparation of probiotics-fermented milk

Briefly, *Lactobacillus casei* ATCC393 was added to sterile MRS broth medium for expanded culture and added to skim milk powder solution (11%) at 4% concentration (1.5×10^8^ CFU/mL) and cultured at 37°C for 24 hours. After the skim milk powder solution changed from liquid to viscous, the solution was centrifuged at 4°C for 10 min at 2500 rpm and the supernatant was taken. The pH of the supernatant was then adjusted to 7.5 with a 10% sodium hydroxide solution.

### Gut microbiota 16sRNA sequencing

After 12 weeks of feeding, feces were collected from all mice using sterile tubes with at least two feces per mouse (>0.1g/serving) and stored immediately at -80°C. Fecal samples from 8 mice in the experimental and control groups were randomly selected for 16S rRNA sequencing using MiSeq technology (26). Briefly, DNA was extracted and the 16S’V4-V5’ region was amplified using specific primers (515F 5’-GTG CCA GCM GCCGCG GTAA-3’; 926R 5’-CCG TCA ATT CMT TTG AGT TT-3’). The amplified products were purified and quantified to create a 16S rRNA library and sequenced (Illumina). Sequences were then quality trimmed to identify and remove chimeric sequences (Trimmomatic and mothur), and sequences were classified using USEARCH software to remove those classified as eukaryotic, archaeal, chloroplast, mitochondrial, or unknown. Finally, the sequence data were filtered to remove any sequences that appeared only once in the dataset, and the clean tags processed above were clustered OTU, and the sequences were clustered to operational taxonomic units (OTUs) with a 97% similarity using USEARCH. The community richness and diversity were analyzed by Mothur to explore the alpha diversity of mouse gut microbiota and the community richness was assessed by ACE estimator and CHO estimator, and the community richness was assessed by Shannon estimator and Simpson estimator. To visually investigate the similarity or difference of the data, the eigenvalues and eigenvectors between samples were determined by calculating ecological distances to perform a principal coordinate analysis based on evolutionary relationships and to create a matrix heat map.

### Bone mineral density measurement

Mice were anesthetized and fixed on a test bench, and bone mineral density of the lumbar spine was measured (scan speed 1 mm/s, resolution 0.5x0.5 mm) using dual-energy X-rays (GE Healthcare, Piscataway, NJ, USA). The instrument was calibrated with a companion model before each measurement. the mean BMD of L3 and L4 was considered to be the BMD of the lumbar spine.

### ELISA

After 8 weeks of antibiotic feeding and 4 weeks after femur fracture surgery, 1 mL of blood was taken from the heart under anesthesia, placed at 4°C overnight, and then centrifuged at 4°C for 10 min at 8000 rpm. The supernatant was extracted and tested for mouse Angiotensin II (Ang II), and Receptor Activator for Nuclear Factor-κ B Ligand (RANKL) ELISA kits (Elabscience Biotechnology Co., Wuhan, Hubei, China) after returning to room temperature according to the manufacturer’s instructions (27).

### Micro-CT

Tibial and femoral samples were collected 4 weeks after femoral fracture surgery, after execution of the mice, the intramedullary pins were removed and immediately placed in 10 formalin for 24 hours for fixation, and the bone was then transferred to a 70% ethanol solution for scanning with a Siemens Inveon Micro-PET/CT system. Voxel resolution was 20 μm. each run included the bones of mice under experimental conditions and calibrated models to standardize grayscale values and maintain consistency between analyses. The growth of the callus at the fracture was assessed by imaging and 3D reconstruction. The bone was also separated from the bone marrow using a fixation threshold (980), and bone mineral density (BMD), trabecular thickness (Tb. Th), trabecular number (Tb. N), and spacing (Tb. Sp) were assessed in the area of secondary trabeculae 1-1.5 mm from the proximal tibial growth plate, using hand-drawn contour lines to identify the area of interest. The data were then analyzed and recorded using Inveon Research Workplace software (Siemens, Germany). Surface images such as femoral trabeculae were taken from an area of the femur with a length of 1.0 mm and a diameter of 1.0 mm for analysis. All bone analyses were performed without regard to experimental conditions.

### Postoperative X-ray examination

At weeks 1, 2, and 4 after femoral fracture surgery, mice were anesthetized with 1% sodium pentobarbital solution and fixed on a test bench, and the growth of bone callus was evaluated using X-ray scans. The scoring criteria used were the Radiological United Score of the Tibial(RUST) (28) fracture healing score as follows: visible fracture line without callus Score = 1; visible fracture line with healing tissue formation Score = 2; no visible fracture line with bridging callus Score = 3. The above criteria were evaluated at the anterior, posterior, medial, and lateral aspects of the fracture site, with a total score of 4-12.

### HE staining of the femur

Four weeks after femoral fracture surgery, femoral specimens were collected after execution of the mice, the intramedullary pins were removed, repeatedly rinsed with saline, and immersed in ethylenediaminetetraacetic acid solution (10%) for one month (sample volume: solution volume >20:1), and the solution was changed every 3 days. The decalcified samples were embedded in paraffin, cut into slices (thickness: 10 μm; 1 slice/sample), and stained with hematoxylin-eosin (H&E). Histological evaluation of stained sections of fracture ends using light microscopy.

### Immunohistochemistry

After preparing the femoral sections using the same procedure as above, the sections were immunohistochemically processed using an anti-alpha smooth muscle actin antibody according to the manufacturer’s instructions (Abcam, United Kingdom). Briefly, sections were washed with TBS solution containing 0.025% Triton X-100 and then closed for 2 h at room temperature using TBS solution containing 10% normal serum and 1% BSA. rabbit antisera against mouse alpha-smooth muscle (Abcam, United Kingdom) applied to sections overnight at 4°C. To visualize the antigen-antibody reaction, the slides were incubated in a TBS solution containing 0.3% H2O2 for 15 min. Finally, sections were counterstained with hematoxylin.

### Statistical analysis

Statistical analysis was performed by using Graph Pad Prism. Measurements that conformed to a normal distribution were compared between two groups using the t-test and between three groups using one-way ANOVA. For measurements that did not conform to a normal distribution, the Wilcoxon test was used to make comparisons between the two groups. Differences of P<0.05 were considered statistically significant.

## Results

### Antibiotic treatment significantly alters the composition of the intestinal microbiota in mice

**Figure 2:**
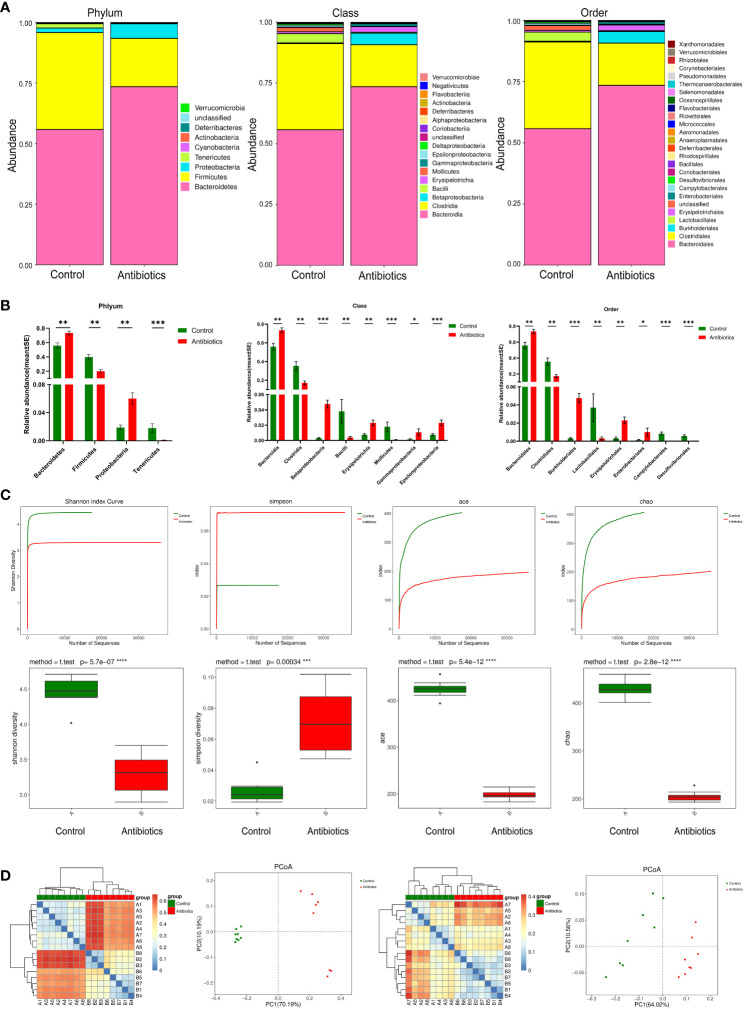
antibiotics altered gut microbiota. **(A)** Comparison of two groups of gut microbiota. **(B)** Difference of gut microbiota between two groups. **(C)** Alpha Diversity Analysis. **(D)** Beta diversity analysis based on evolutionary relationships, matrix heat map and POCA analysis of Weightedunifrac and unweightedunifrac. *p<0.05, **p<0.01, ***p<0.001, ****p<0.0001.

To investigate the effect of antibiotic treatment on the gut microbiota of mice, we performed 16S RNA sequencing of fecal samples to analyze the composition of the mouse gut microbiota at three levels: phylum, class, and order (**Figure 2**). The results showed that after 12 weeks of antibiotic treatment, profound changes in the composition of the gut microbiota occurred at all three levels. Compared with the control group, the antibiotic group showed a significant increase in the relative abundance of *Bacteroidetes* phylum and a significant decrease in the relative abundance of *Firmicutes* phylum at the phylum level, and an increase in the relative abundance of *Bacteroidales* and a decrease in the relative abundance of *Clostridiales* at the class level, as well as an increase in the relative abundance of *Bacteroidales* and a decrease in the relative abundance of *Clostridiales* at the order level. (**Figure 2A**). The non-parametric test results also showed that the above populations were the most diverse at their corresponding levels (**Figure 2B**).To further investigate the effect of antibiotic treatment on the species richness of the gut microbiota, we used the Shannon, Simpson, Ace, and Chao estimators to explore the alpha diversity of the mouse GM, and the results showed that antibiotic treatment significantly reduced the Shannon, Ace and Chao indices, while the Simpson index was significantly increased, suggesting that antibiotics treatment significantly decreased the species richness of mouse GM (**Figure 2C**). In addition, we also multi-beta diversity was analyzed, as shown in **Figure 2D** The thermal matrix plot and PCoA analysis both showed dispersed samples from different groups and concentrated samples from the same group. In conclusion, the above results suggest that antibiotic treatment significantly altered the composition and reduced the complexity of the gut microbiota in mice.

### Antibiotic treatment leads to the development of osteoporosis in mice

**Figure 3:**
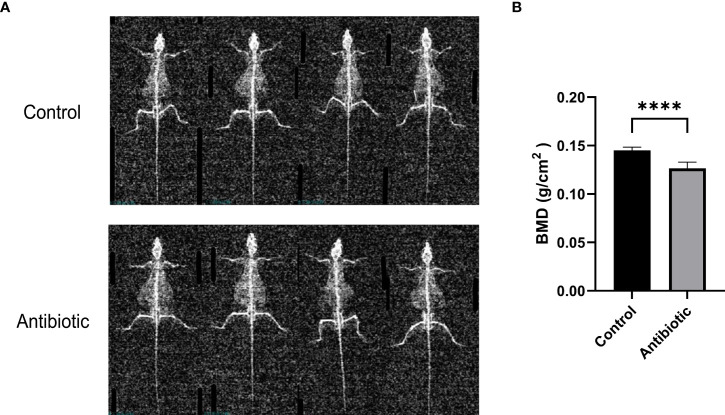
Dual-energy X-ray images and quantification of bone mineral density were analyzed **(A)** dual-energy X-ray images of the control and antibiotic groups; **(B)** comparison of bone mineral density between the two groups (P<0.0001). Data points are expressed as mean ± SD, and a t-test was used for comparison between groups. ****p<0.0001.

To investigate the effect of antibiotic treatment on bone quality in mice, we assessed bone mineral density (BMD) of the spine using dual-energy X-rays. Dual-energy radiographs showed that the grayness of the spine was significantly lower in antibiotic-treated mice than in controls (**Figure 3A**). Correspondingly, the quantitative analysis of L3-L4 showed a significant decrease in BMD in the antibiotic group compared to the control group (**Figure 3B**).

### Antibiotic treatment leads to increased Ang II and RANKL in mice serum

**Figure 4:**
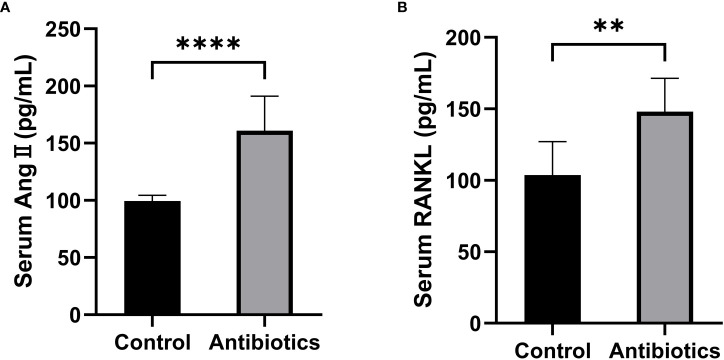
Comparison of serum Ang II and RANKL levels between the control group and the antibiotic group. **(A)** Serum angiotensin II (Ang II); **(B)** Receptor Activator for Nuclear Factor-κ B Ligand (RANKL). **p<0.01, ***p<0.001.

To investigate whether antibiotic-induced osteoporosis is related to RAS, we measured the serum RAS fractions and RANKL levels in mice after 12 weeks of antibiotic treatment. The results showed that Ang II and RANKL levels were significantly increased in the serum of mice in the antibiotic group, suggesting that osteoporosis caused by antibiotic treatment may be produced by activating the RAS (**Figure 4**).

### *Lactobacillus casei* fermented milk may improve fracture healing in osteoporotic mice

**Figure 5:**
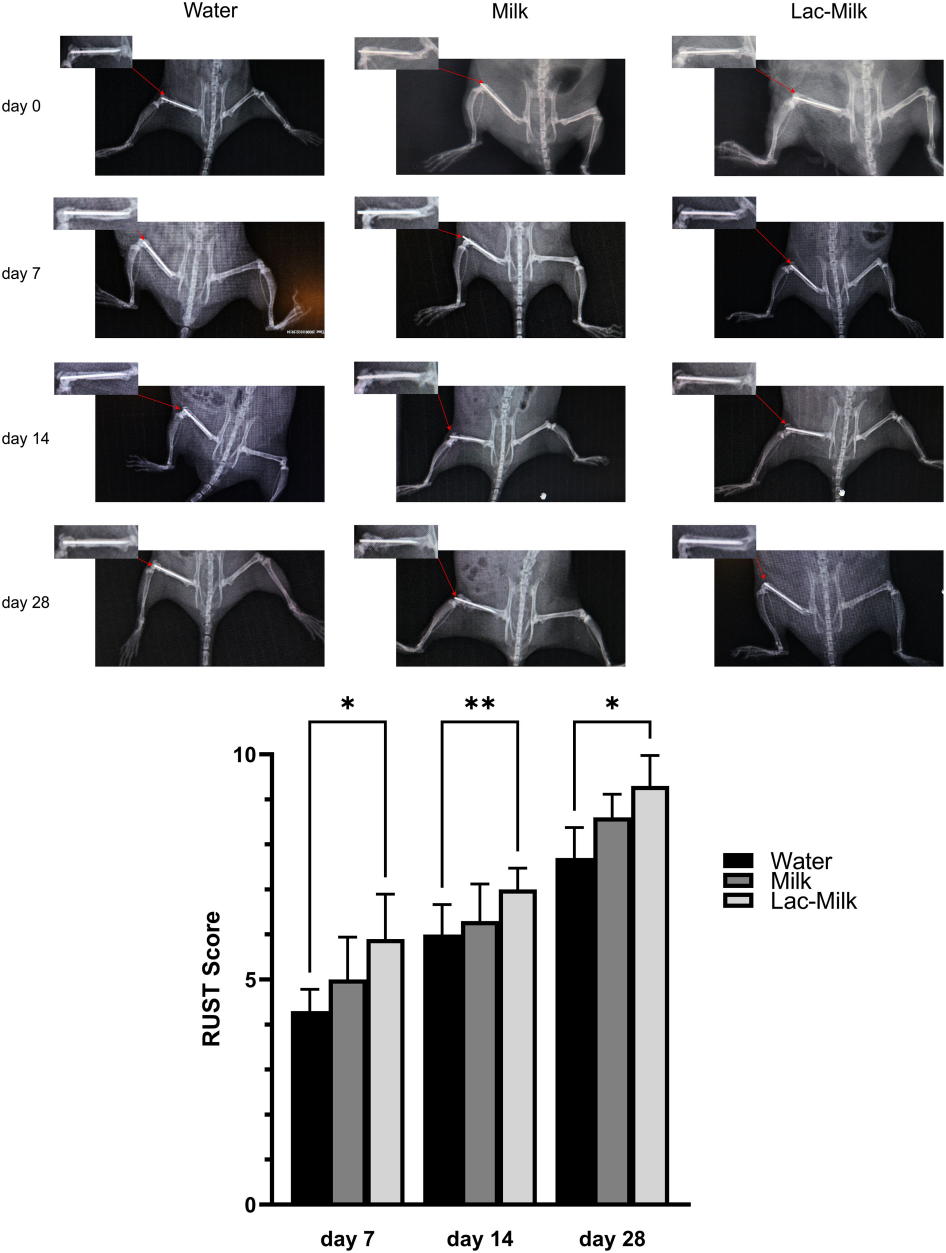
Postoperative X-ray images and RUST scores of femoral fractures. **(A)** Representative images at 1, 7, 14, and 28 days postoperatively. **(B)** RUST scores at 7, 14, and 28 days postoperatively. *p<0.05, **p<0.01.

**Figure 6:**
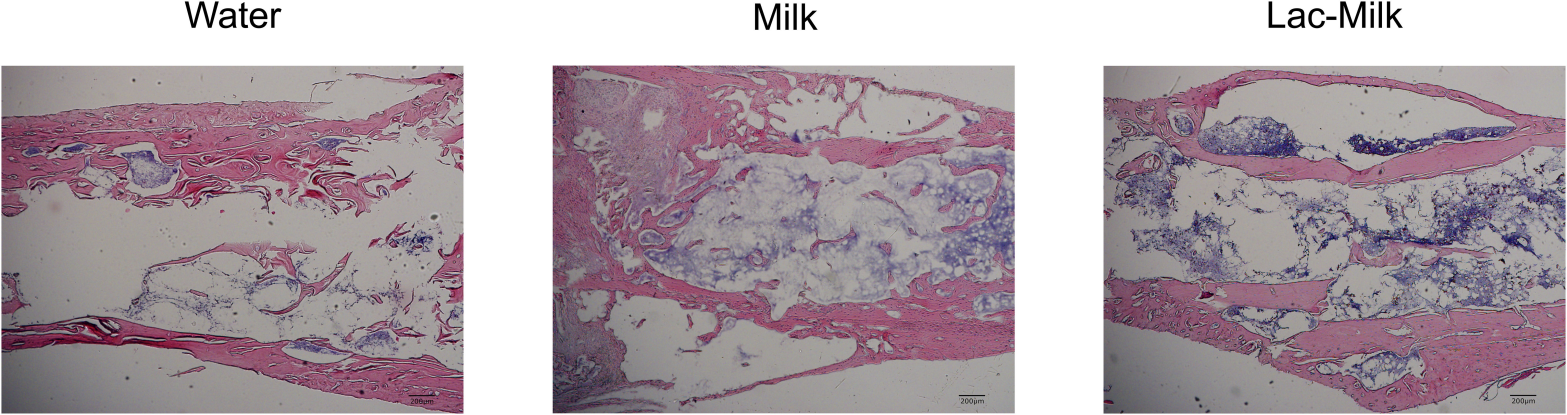
Representative HE stained images of the fracture site 28 days after surgery.

To investigate the effect of *Lactobacillus casei* fermented milk on fracture healing in osteoporotic mice, we performed X-ray scans on mice at 1, 7, 14, and 28 days after surgery to assess postoperative fracture healing. As shown in **Figure 5A**, the fracture alignment was satisfactory and there was no obvious displacement of the intramedullary pin on postoperative day 1; on postoperative day 7, the fracture line was visible and healing tissue formation was visible in some mice, but no obvious callus growth was observed; on a postoperative day 14, the fracture line was still clearly visible and callus formation was observed in the stress measurement, and callus formation was significantly more in the *Lactobacillus casei* fermented milk group; on postoperative day 28, the fracture line was slightly blurred and the callus formation was significantly increased. Significant callus formation was observed in the milk and *Lactobacillus casei* fermented milk groups on both the stress side and the tension side, but more pronounced callus formation was observed in the water group only on the stress side. We quantitatively assessed the fracture healing using the RUST scale, and the results showed that the fracture healing was significantly better in the *Lactobacillus casei* fermented milk group than in the water group at all time points (**Figure 5B**). In addition, we performed the histological assessment of fracture healing in mice by HE staining, as shown in **Figure 6**. The bone trabeculae in the callus at the fracture in the water group were sparsely and irregularly arranged, those in the callus in the milk group were slightly dense and regular, and the fibrous callus in the *Lactobacillus casei* fermented milk group disappeared and the bone marrow cavity was partially recanalized, which was significantly better than the water and milk groups.

### *Lactobacillus casei* fermented milk improves bone microstructure and promotes callus formation

**Figure 7:**
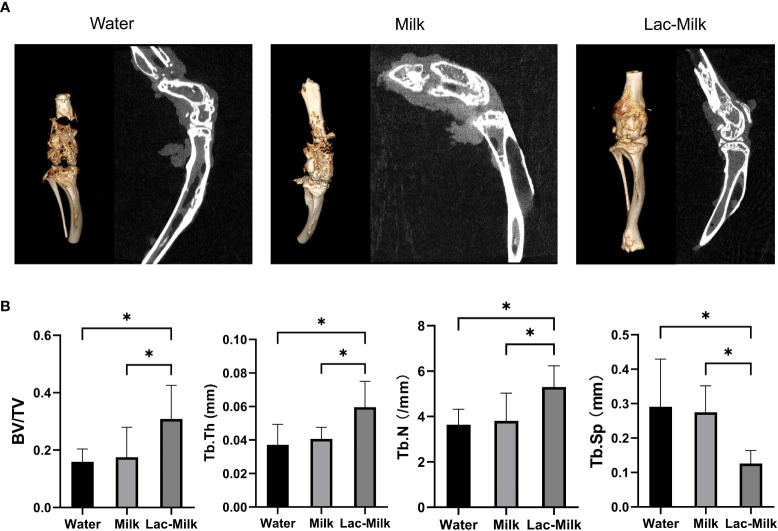
Micro-CT images and microparametric analysis of tibial trabeculae at 28 days postoperatively. **(A)** Representative Micro-CT images. **(B)** Quantitative analysis of bone trabecular number (Tb. N), bone value/total value (BV/TV), bone trabecular thickness (Tb. Th), and bone trabecular space (Tb. Sp). *p<0.05.

To further investigate the effect of *Lactobacillus casei* on fracture healing, we performed Micro-CT scans of the tibia and femur of mice at 28 days postoperatively and performed 3D reconstruction to assess callus growth and bone trabecular microparameters. The results showed that only a few calluses formed at the fracture in the water group and partial separation of the fracture were observed, whereas the calluses at the fracture in the mice in the *Lactobacillus casei* fermented milk group were significantly more numerous and connected into pieces, which was significantly better than the milk and water groups (**Figure 7A**). In addition, the evaluation of tibial trabecular parameters in mice showed that the BV/TV, Tb. Th, and Tb. N of mice in the *Lactobacillus casei* fermented milk group was significantly higher than those in the water and milk groups, and the corresponding Tb. Sp was significantly lower than that in the water and milk groups(**Figure 7B**).

### *Lactobacillus casei* fermented milk significantly increased the expression of α smooth muscle actin in the callus

**Figure 8:**
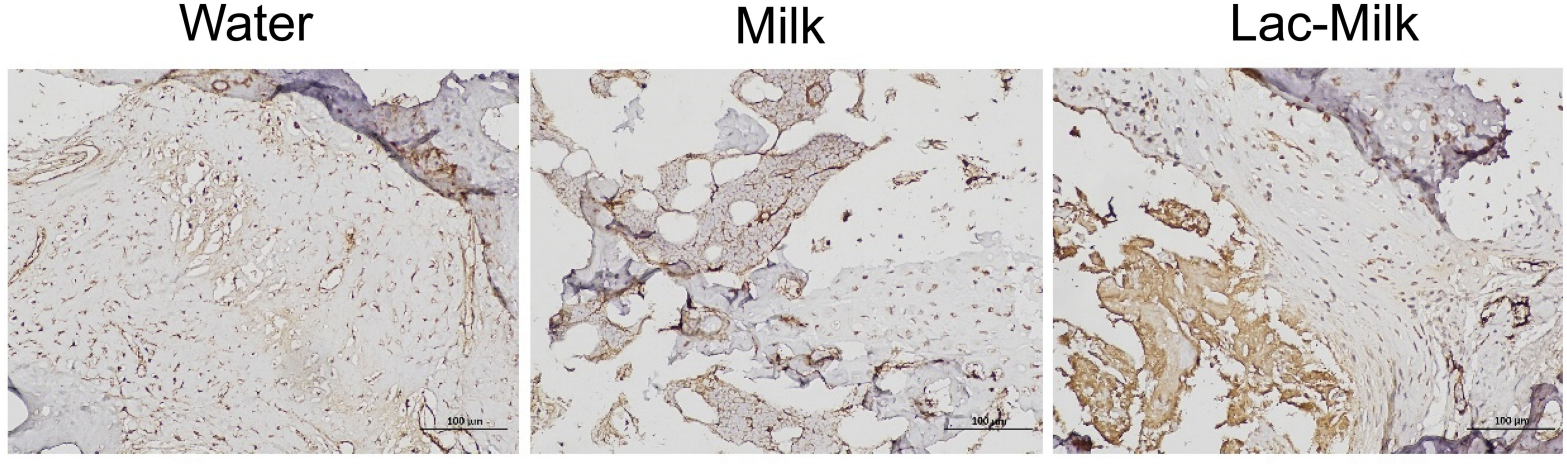
Representative immunohistochemical staining images (400×).

α smooth muscle actin (αSMA), a specific marker of osteochondral progenitor cells during fracture healing, is one of the important indicators for assessing fracture healing (29). We explored its expression in callus by immunohistochemistry, and the results showed that αSMA expression was significantly higher in bone scabs of mice in the *Lactobacillus casei* fermented milk group compared to the water and milk groups (**Figure 8**).

### *Lactobacillus casei* fermented milk reduces Ang II and RANKL levels in mice serum

**Figure 9:**
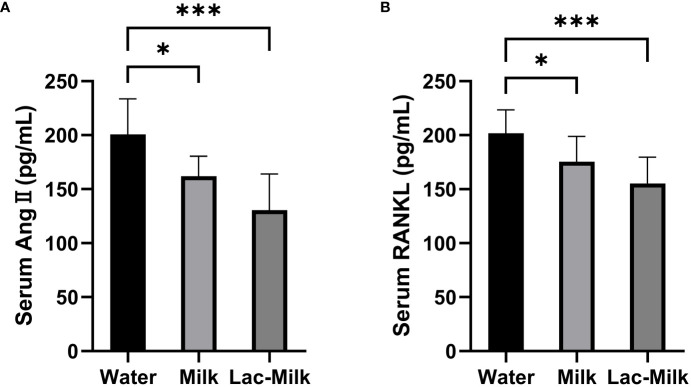
Comparison of serum Ang II and RANKL levels in the water and milk groups with the *Lactobacillus casei* fermented milk group. **(A)** Serum angiotensin II (Ang II); **(B)** Receptor Activator for Nuclear Factor-κ B Ligand (RANKL). *p<0.05, ***p<0.001.

To investigate whether the osteoprotective effect of *Lactobacillus casei* fermented milk was related to RAS, we measured the serum levels of Ang II and RANKL in mice 28 days after surgery. The results showed that the serum levels of Ang II and RANKL were significantly lower in the *Lactobacillus casei* fermented milk group compared with the water and milk groups. It is suggested that the osteoprotective effect of *Lactobacillus casei* fermented milk may be related to the inhibition of RAS activity (**Figure 9**).

## Discussion

Our study showed that antibiotic treatment for 12 weeks led to significant changes in the gut microbiota and osteoporosis in mice, and the levels of Ang II and RANKL in serum were significantly increased, suggesting that RAS may play an important role in bone loss due to gut microbiota dysbiosis. And that *Lactobacillus casei* fermented milk treatment alleviated osteoporosis and promoted fracture healing in mice, and that this effect may have been produced by inhibiting Ang II and RANKL production.

The gut microbiota has recently been shown to affect multiple systems in the host, including the skeleton (1–5). Studies on germ-free mice have shown the importance of gut microbiota for bone metabolism, but the problem of defective immune system development in germ-free mice cannot be avoided (5). Therefore, perturbing the gut microbiota through antibiotic therapy may be a better option (30–33) It is well known that antibiotics are widely used to treat and prevent bacterial infections. However, it has been shown that antibiotic treatment can have lasting negative effects on the host by depleting commensal bacteria leading to dysbiosis and long-term changes in the gut microbiota (34–36). This provides an effective means for exploring the link between gut microbiota and bone metabolism. However, the gut microbiota is usually in a dynamic homeostatic transient state, with strong resistance to external influences. Studies have shown that while short-term antibiotic treatment (1-2 weeks) can perturb the gut microbiota, the gut microbiota can quickly return to its pre-treatment state after antibiotic treatment is discontinued (37). For this reason, our study used neomycin and ampicillin for a longer-term (12 weeks) antibiotic treatment to cause long-term and sustained disruption of the gut microbiota (38). To effectively limit the parenteral effects of antibiotic treatment, two antibiotics, neomycin, and ampicillin, with low intestinal absorption in rodents, were used in this study to minimize parenteral effects (39, 40). Although it is rare for a human to be treated with antibiotics throughout growth and development, there are cases of long-term and significant changes in the gut microbiota due to diet or metabolism (37).

Previous studies have demonstrated that antibiotic treatment can have a significant effect on the gut microbiota (30–33), and our findings similarly demonstrate this. Comparing the differences in gut microbiota structure between the antibiotic-treated and control groups by 16s RNA sequencing, our results showed significant alterations in the gut microbiota of mice after antibiotic treatment at multiple levels from phylum to order. Previous studies have shown that antibiotic treatment can cause a decrease in the abundance of the phylum *Bacteroidetes*, a major phylum in healthy mice and humans and that a decrease in the abundance of *Bacteroidetes* is associated with many diseases, including IBD and type 2 diabetes (41, 42). And interestingly, in our study, alterations in the gut microbiota of mice after antibiotic treatment were mainly manifested at the phylum level by an increase in the relative abundance of the *Bacteroidetes* phylum and a decrease in the relative abundance of the *Firmicutes* phylum. We note the presence of *Lactobacillus*, a widely studied and used bacterium in the *Firmicutes* phylum, and studies have shown that *Lactobacillus* treatment is beneficial in increasing bone mineral density (7, 10, 43). And the relative abundance of *Lactobacillus* also showed a significant decrease in our study. Therefore, we speculate that the decrease in the relative abundance of *Lactobacillus* may be one of the reasons for bone loss due to antibiotic treatment. However, it is noteworthy that the altered abundance of the *Bacteroidetes* phylum in our study appeared significantly different from previous studies, which we believe may be related to the type of antibiotic, dose, duration of treatment, strain, sex, and survival environment of the mice.

Many studies have shown that the gut microbiota is critical to bone metabolism and that dysbiosis of the gut microbiota can lead to bone loss (6). This was also demonstrated in our study, where antibiotic treatment resulted in a significant reduction in spinal bone mineral density in mice. Although antibiotic therapy has been widely used to explore the link between gut microbiota and bone metabolism, we note that many studies have shown mixed results. For example, female mice (C57BL/6J) showed a significant increase in BMD after 3 weeks of antibiotic treatment, but no effect after 7 weeks (30); Low doses of penicillin increased BMD in female mice, while males receiving the same treatment showed decreased BMD (44). In conclusion, antibiotic treatment can affect bone metabolism, but the effect depends on the duration of antibiotic treatment as well as the age, sex, and strain of the mice.

Oral probiotics are the most common treatment for gut microbiota dysbiosis, and many studies have demonstrated that probiotic therapy can alleviate multiple factors contributing to osteoporosis (7, 8, 45). *Lactobacillus* is the most widely studied and used, and is available through diet (e.g., yogurt). Therefore, for our study, we chose to use *Lactobacillus casei* ATCC393 for the treatment of osteoporotic mice after dysbiosis, and in addition, we added *Lactobacillus casei* to skim milk to make *Lactobacillus casei* fermented milk to imitate the most common way of obtaining *Lactobacillus* in humans. Our study showed that *Lactobacillus casei* fermented milk treatment significantly improved the microstructure of bone trabeculae in osteoporotic mice caused by dysbiosis of the gut microbiota, furthermore, it had a significant effect on the healing of fractures in osteoporotic mice. Our data showed that BV/TV, Tb. Th and Tb. N was significantly higher and Tb. Sp was significantly lower in mice treated with *Lactobacillus casei* fermented milk, and histomorphology and imaging showed significantly faster and more callus formation and significantly faster healing at the femur fracture. And the expression of αSMA in callus was significantly increased in mouse callus after *Lactobacillus casei* fermented milk treatment.

To investigate the possible mechanism of gut microbiota affecting bone metabolism in this study, we measured the levels of Ang II and RANKL in mice serum, and the results showed that the levels of Ang II and RANKL in mice serum were significantly increased after antibiotic treatment, while *Lactobacillus casei* fermented milk treatment significantly decreased the levels of Ang II and RANKL in serum. Ang II acts as a major effector protein of RAS, and our results suggest that RAS may have an important role in osteoporosis caused by dysbiosis of the gut microbiota. Tissue localized RAS is called tissue RAS (46), studies have shown that the local RAS is thought to be involved in a variety of physiological and pathological processes, such as insulin secretion, glomerulosclerosis, nephritis, and atherosclerosis (47–50). Recent studies have found that RAS components such as ACE and Ang II receptors are expressed in the local environment of bone tissue and callus (19–21). Many studies have shown that local RAS activation was found to be involved in age-related osteoporosis in mice (22), onset and development of postmenopausal osteoporosis in an ovariectomy model (23, 24), and osteoporosis due to overuse of glucocorticoids (20), suggesting an important role for RAS in the development of osteoporosis. In addition, Shimizu (23) and Zhou (24) found that Ang II promotes osteoclast proliferation and differentiation by inducing RANKL release from osteoblasts, which in turn leads to osteoporosis. Our results also show that alterations in the gut microbiota result in significant changes in serum Ang II and RANKL with the same trend and, more importantly, that these changes can affect bone mineral density and fracture healing rate. This suggests that RAS may be one of the important pathways through which the gut microbiota affects bone metabolism. We note that studies have shown that *Lactobacillus* fermented milk produces valinyl-prolinyl-proline (VPP) and the bioactive peptide isoleucine-prolinyl-proline (IPP), and in addition, these small peptides have angiotensin-converting enzyme (ACE) inhibitory activity that blocks the conversion of Ang I to Ang II and inhibits the breakdown of bradykinin by inhibiting ACE (16–18). We believe that this may be one of the reasons why gut microbiota affects RAS. And in our study, we found a significant reduction of *Lactobacillus* with osteoporosis after antibiotic treatment, while *Lactobacillus casei* fermented milk treatment resulted in significant improvement of bone trabecular microparameters, alleviation of osteoporosis, and a significant acceleration of fracture healing, which seems to corroborate this view, but further studies are needed. And in our study, we found a significant reduction of *Lactobacillus* with osteoporosis after antibiotic treatment, while *Lactobacillus casei* fermented milk treatment resulted in significant improvement of bone trabecular microparameters, alleviation of osteoporotic symptoms, and a significant acceleration of fracture healing, which seems to corroborate this view, but further studies are needed.

In conclusion, our study shows that antibiotic treatment leads to significant alterations in the gut microbiota and causes the development of osteoporosis in mice, that *Lactobacillus casei* fermented milk treatment alleviates this osteoporosis and accelerates fracture healing, and that this effect is produced by modulating RAS activity and thus affecting RANKL release. However, this study has several limitations. First, only one concentration of antibiotic treatment was studied, which does not adequately determine the relationship between antibiotic treatment and bone loss and RAS. Second, our results contradict previous studies. The effect of antibiotic treatment on the gut microbiota still needs further study. Third, although 16s RNA sequencing of fecal specimens is currently the predominant way to assess the composition of the gut microbiota, there is a possibility that the fecal microbiota does not reflect the entire gut microbial environment. In addition, we only examined the circulating RAS fractions and RANKL in mice and did not perform the local examination of bone tissue. Therefore, our findings still require further studies to better elucidate the link between gut microbiota and bone metabolism.

## Data availability statement

The original contributions presented in the study are included in the article/supplementary material. Further inquiries can be directed to the corresponding author.

## Ethics statement

The animal study was reviewed and approved by Animal Research and Care Committee of Southwestern Medical University (NO. SWMU20220091).

## Author contributions

XG, KZ, LZ, contributed equally to this study. All authors contributed to the article and approved the submitted version.

## Conflict of interest

The authors declare that the research was conducted in the absence of any commercial or financial relationships that could be construed as a potential conflict of interest.

## Publisher’s note

All claims expressed in this article are solely those of the authors and do not necessarily represent those of their affiliated organizations, or those of the publisher, the editors and the reviewers. Any product that may be evaluated in this article, or claim that may be made by its manufacturer, is not guaranteed or endorsed by the publisher.

## References

[1] Collins SM. A role for the gut microbiota in ibs. Nat Rev Gastroenterol Hepatol (2014) 11(8):497–505. doi: 10.1038/nrgastro.2014.40 24751910

[2] Ley RE Hamady M Lozupone C Turnbaugh PJ Ramey RR Bircher JS. Evolution of mammals and their gut microbes. Sci (New York NY) (2008) 320(5883):1647–51. doi: 10.1126/science.1155725 PMC2649005 18497261

[3] Tremaroli V Bäckhed F. Functional interactions between the gut microbiota and host metabolism. Nature (2012) 489(7415):242–9. doi: 10.1038/nature11552 22972297

[4] Belkaid Y Hand TW. Role of the microbiota in immunity and inflammation. Cell (2014) 157(1):121–41. doi: 10.1016/j.cell.2014.03.011 PMC4056765 24679531

[5] Sjögren K Engdahl C Henning P Lerner UH Tremaroli V Lagerquist MK. The gut microbiota regulates bone mass in mice. J Bone Mineral Res (2012) 27(6):1357–67. doi: 10.1002/jbmr.1588 PMC3415623 22407806

[6] Irwin R Lee T Young VB Parameswaran N McCabe LR. Colitis-induced bone loss is gender dependent and associated with increased inflammation. Inflammation Bowel Dis (2013) 19(8):1586–97. doi: 10.1097/MIB.0b013e318289e17b PMC4127911 23702805

[7] Ohlsson C Engdahl C Fåk F Andersson A Windahl SH Farman HH. Probiotics protect mice from ovariectomy-induced cortical bone loss. PloS One (2014) 9(3):e92368. doi: 10.1371/journal.pone.0092368 24637895 PMC3956931

[8] Britton RA Irwin R Quach D Schaefer L Zhang J Lee T. Probioticl. reuteritreatment prevents bone loss in a menopausal ovariectomized mouse model. J Cell Physiol (2014) 229(11):1822–30. doi: 10.1002/jcp.24636 PMC4129456 24677054

[9] Zhang J Motyl KJ Irwin R MacDougald OA Britton RA McCabe LR. Loss of bone and Wnt10b expression in Male type 1 diabetic mice is blocked by the probiotic lactobacillus reuteri. Endocrinology (2015) 156(9):3169–82. doi: 10.1210/en.2015-1308 PMC4541610 26135835

[10] Schepper JD Collins F Rios-Arce ND Kang HJ Schaefer L Gardinier JD. Involvement of the gut microbiota and barrier function in glucocorticoid-induced osteoporosis. J Bone Mineral Res (2020) 35(4):801–20. doi: 10.1002/jbmr.3947 31886921

[11] Li J-Y Chassaing B Tyagi AM Vaccaro C Luo T Adams J. Sex steroid deficiency–associated bone loss is microbiota dependent and prevented by probiotics. J Clin Invest (2016) 126(6):2049–63. doi: 10.1172/jci86062 PMC4887186 27111232

[12] Garcia VG Knoll LR Longo M Novaes VC Assem NZ Ervolino E. Effect of the probiotic saccharomyces cerevisiae on ligature-induced periodontitis in rats. J Periodontal Res (2016) 51(1):26–37. doi: 10.1111/jre.12274 25918871

[13] Bayat M Dabbaghmanesh MH Koohpeyma F Mahmoodi M Montazeri-Najafabady N Bakhshayeshkaram M. The effects of soy milk enriched with lactobacillus casei and omega-3 on the tibia and L5 vertebra in diabetic rats: A stereological study. Probiotics Antimicrob Proteins (2018) 11(4):1172–81. doi: 10.1007/s12602-018-9482-z 30406893

[14] Pan H Guo R Ju Y Wang Q Zhu J Xie Y. A single bacterium restores the microbiome dysbiosis to protect bones from destruction in a rat model of rheumatoid arthritis. Microbiome (2019) 7(1):107. doi: 10.1186/s40168-019-0719-1 31315667 PMC6637628

[15] Kim JG Lee E Kim SH Whang KY Oh S Imm J-Y. Effects of a lactobacillus casei 393 fermented milk product on bone metabolism in ovariectomised rats. Int Dairy J (2009) 19(11):690–5. doi: 10.1016/j.idairyj.2009.06.009

[16] Narva M Collin M Lamberg-Allardt C Karkkainen M Poussa T Vapaatalo H. Effects of long-term intervention with lactobacillus helveticus-fermented milk on bone mineral density and bone mineral content in growing rats. Ann Nutr Metab (2004) 48(4):228–34. doi: 10.1159/000080455 15334032

[17] Narva M Halleen J Vaananen K Korpela R. Effects of lactobacillus helveticus fermented milk on bone cells *in vitro* . Life Sci (2004) 75(14):1727–34. doi: 10.1016/j.lfs.2004.04.011 15268972

[18] Narva M Rissanen J Halleen J Vapaatalo H Vaananen K Korpela R. Effects of bioactive peptide, valyl-Prolyl-Proline (Vpp), and lactobacillus helveticus fermented milk containing vpp on bone loss in ovariectomized rats. Ann Nutr Metab (2007) 51(1):65–74. doi: 10.1159/000100823 17356257

[19] Izu Y Mizoguchi F Kawamata A Hayata T Nakamoto T Nakashima K. Angiotensin ii type 2 receptor blockade increases bone mass. J Biol Chem (2009) 284(8):4857–64. doi: 10.1074/jbc.M807610200 PMC2742875 19004830

[20] Yongtao Z Kunzheng W Jingjing Z Hu S Jianqiang K Ruiyu L. Glucocorticoids activate the local renin–angiotensin system in bone: Possible mechanism for glucocorticoid-induced osteoporosis. Endocrine (2014) 47(2):598–608. doi: 10.1007/s12020-014-0196-z 24519760

[21] Garcia P Schwenzer S Slotta JE Scheuer C Tami AE Holstein JH. Inhibition of angiotensin-converting enzyme stimulates fracture healing and periosteal callus formation - role of a local renin-angiotensin system. Br J Pharmacol (2010) 159(8):1672–80. doi: 10.1111/j.1476-5381.2010.00651.x PMC2925490 20233225

[22] Gu S-s Zhang Y Li X-l Wu S-y Diao T-y Hai R. Involvement of the skeletal renin-angiotensin system in age-related osteoporosis of ageing mice. Biosci Biotechnol Biochem (2014) 76(7):1367–71. doi: 10.1271/bbb.120123 22785482

[23] Shimizu H Nakagami H Osako MK Hanayama R Kunugiza Y Kizawa T. Angiotensin ii accelerates osteoporosis by activating osteoclasts. FASEB J (2008) 22(7):2465–75. doi: 10.1096/fj.07-098954 18256306

[24] Zhou Y Guan X Chen X Yu M Wang C Chen X. Angiotensin Ii/Angiotensin ii receptor blockade affects osteoporosis *Via* the At1/At2-mediated camp-dependent pka pathway. Cells Tissues Organs (2017) 204(1):25–37. doi: 10.1159/000464461 28478436

[25] Donmez BO Ozdemir S Sarikanat M Yaras N Koc P Demir N. Effect of angiotensin ii type 1 receptor blocker on osteoporotic rat femurs. Pharmacol Rep (2012) 64(4):878–88. doi: 10.1016/s1734-1140(12)70882-4 23087139

[26] Caporaso JG Lauber CL Walters WA Berg-Lyons D Huntley J Fierer N. Ultra-High-Throughput microbial community analysis on the illumina hiseq and miseq platforms. ISME J (2012) 6(8):1621–4. doi: 10.1038/ismej.2012.8 PMC3400413 22402401

[27] Engvall E. The Elisa, enzyme-linked immunosorbent assay. Clin Chem (2010) 56(2):319–20. doi: 10.1373/clinchem.2009.127803 19850633

[28] Leow JM Clement ND Tawonsawatruk T Simpson CJ Simpson AH. The radiographic union scale in tibial (Rust) fractures: Reliability of the outcome measure at an independent centre. Bone Joint Res (2016) 5(4):116–21. doi: 10.1302/2046-3758.54.2000628 PMC5009237 27073210

[29] Matthews BG Torreggiani E Roeder E Matic I Grcevic D Kalajzic I. Osteogenic potential of alpha smooth muscle actin expressing muscle resident progenitor cells. Bone (2016) 84:69–77. doi: 10.1016/j.bone.2015.12.010 26721734 PMC4755912

[30] Cho I Yamanishi S Cox L Methé BA Zavadil J Li K. Antibiotics in early life alter the murine colonic microbiome and adiposity. Nature (2012) 488(7413):621–6. doi: 10.1038/nature11400 PMC3553221 22914093

[31] Guss JD Horsfield MW Fontenele FF Sandoval TN Luna M Apoorva F. Alterations to the gut microbiome impair bone strength and tissue material properties. J Bone Mineral Res (2017) 32(6):1343–53. doi: 10.1002/jbmr.3114 PMC5466506 28244143

[32] Hathaway-Schrader JD Steinkamp HM Chavez MB Poulides NA Kirkpatrick JE Chew ME. Antibiotic perturbation of gut microbiota dysregulates osteoimmune cross talk in postpubertal skeletal development. Am J Pathol (2019) 189(2):370–90. doi: 10.1016/j.ajpath.2018.10.017 PMC6360355 30660331

[33] Rios-Arce ND Schepper JD Dagenais A Schaefer L Daly-Seiler CS Gardinier JD. Post-antibiotic gut dysbiosis-induced trabecular bone loss is dependent on lymphocytes. Bone (2020) 134:115269. doi: 10.1016/j.bone.2020.115269 32061677 PMC7138712

[34] Scott NA Andrusaite A Andersen P Lawson M Alcon-Giner C Leclaire C. Antibiotics induce sustained dysregulation of intestinal T cell immunity by perturbing macrophage homeostasis. Sci Transl Med (2018) 10(464):eaao4755. doi: 10.1126/scitranslmed.aao4755 30355800 PMC6548564

[35] Zarrinpar A Chaix A Xu ZZ Chang MW Marotz CA Saghatelian A. Antibiotic-induced microbiome depletion alters metabolic homeostasis by affecting gut signaling and colonic metabolism. Nat Commun (2018) 9(1):2872. doi: 10.1038/s41467-018-05336-9 30030441 PMC6054678

[36] Ubeda C Pamer EG. Antibiotics, microbiota, and immune defense. Trends Immunol (2012) 33(9):459–66. doi: 10.1016/j.it.2012.05.003 PMC3427468 22677185

[37] Lozupone CA Stombaugh JI Gordon JI Jansson JK Knight R. Diversity, stability and resilience of the human gut microbiota. Nature (2012) 489(7415):220–30. doi: 10.1038/nature11550 PMC3577372 22972295

[38] Laukens D Brinkman BM Raes J De Vos M Vandenabeele P. Heterogeneity of the gut microbiome in mice: Guidelines for optimizing experimental design. FEMS Microbiol Rev (2016) 40(1):117–32. doi: 10.1093/femsre/fuv036 PMC4703068 26323480

[39] Vijay-Kumar M Aitken JD Carvalho FA Cullender TC Mwangi S Srinivasan S. Metabolic syndrome and altered gut microbiota in mice lacking toll-like receptor 5. Sci (New York NY) (2010) 328(5975):228–31. doi: 10.1126/science.1179721 PMC4714868 20203013

[40] MacGregor RR Graziani AL. Oral administration of antibiotics: A rational alternative to the parenteral route. Clin Infect Dis (1997) 24(3):457–67. doi: 10.1093/clinids/24.3.457 9114201

[41] Tamboli CP Neut C Desreumaux P Colombel JF. Dysbiosis in inflammatory bowel disease. Gut (2004) 53(1):1–4. doi: 10.1136/gut.53.1.1 14684564 PMC1773911

[42] Larsen N Vogensen FK van den Berg FW Nielsen DS Andreasen AS Pedersen BK. Gut microbiota in human adults with type 2 diabetes differs from non-diabetic adults. PloS One (2010) 5(2):e9085. doi: 10.1371/journal.pone.0009085 20140211 PMC2816710

[43] Li JY Chassaing B Tyagi AM Vaccaro C Luo T Adams J. Sex steroid deficiency-associated bone loss is microbiota dependent and prevented by probiotics. J Clin Invest (2016) 126(6):2049–63. doi: 10.1172/jci86062 PMC4887186 27111232

[44] Cox LM Yamanishi S Sohn J Alekseyenko AV Leung JM Cho I. Altering the intestinal microbiota during a critical developmental window has lasting metabolic consequences. Cell (2014) 158(4):705–21. doi: 10.1016/j.cell.2014.05.052 PMC4134513 25126780

[45] Li JY Yu M Pal S Tyagi AM Dar H Adams J. Parathyroid hormone-dependent bone formation requires butyrate production by intestinal microbiota. J Clin Invest (2020) 130(4):1767–81. doi: 10.1172/jci133473 PMC7108906 31917685

[46] Skov J Persson F Frøkiær J Christiansen JS. Tissue renin-angiotensin systems: A unifying hypothesis of metabolic disease. Front Endocrinol (Lausanne) (2014) 5:23. doi: 10.3389/fendo.2014.00023 24592256 PMC3938116

[47] Lau T Carlsson PO Leung PS. Evidence for a local angiotensin-generating system and dose-dependent inhibition of glucose-stimulated insulin release by angiotensin ii in isolated pancreatic islets. Diabetologia (2004) 47(2):240–8. doi: 10.1007/s00125-003-1295-1 14722647

[48] Zhang Z Zhang Y Ning G Deb DK Kong J Li YC. Combination therapy with At1 blocker and vitamin d analog markedly ameliorates diabetic nephropathy: Blockade of compensatory renin increase. Proc Natl Acad Sci U.S.A. (2008) 105(41):15896–901. doi: 10.1073/pnas.0803751105 PMC2562415 18838678

[49] Zhang Y Deb DK Kong J Ning G Wang Y Li G. Long-term therapeutic effect of vitamin d analog doxercalciferol on diabetic nephropathy: Strong synergism with At1 receptor antagonist. Am J Physiol Renal Physiol (2009) 297(3):F791–801. doi: 10.1152/ajprenal.00247.2009 PMC2739712 19535571

[50] Koïtka A Cao Z Koh P Watson AM Sourris KC Loufrani L. Angiotensin ii subtype 2 receptor blockade and deficiency attenuate the development of atherosclerosis in an apolipoprotein e-deficient mouse model of diabetes. Diabetologia (2010) 53(3):584–92. doi: 10.1007/s00125-009-1619-x 19957160

